# Analysis of Immune-Related Signatures Related to CD4+ T Cell Infiltration With Gene Co-Expression Network in Pancreatic Adenocarcinoma

**DOI:** 10.3389/fonc.2021.674897

**Published:** 2021-07-23

**Authors:** Zhen Tan, Yubin Lei, Bo Zhang, Si Shi, Jiang Liu, Xianjun Yu, Jin Xu, Chen Liang

**Affiliations:** ^1^ Department of Pancreatic Surgery, Fudan University Shanghai Cancer Center, Shanghai, China; ^2^ Department of Oncology, Shanghai Medical College, Fudan University, Shanghai, China; ^3^ Shanghai Pancreatic Cancer Institute, Shanghai, China; ^4^ Pancreatic Cancer Institute, Fudan University, Shanghai, China

**Keywords:** pancreatic ductal adenocarcinoma, immunocytes infiltration, CIBERSORT, WGCNA, bioinformatics

## Abstract

**Background:**

Pancreatic ductal adenocarcinoma (PDAC) is one of the most invasive solid malignancies. Immunotherapy and targeted therapy confirmed an existing certain curative effect in treating PDAC. The aim of this study was to develop an immune-related molecular marker to enhance the ability to predict Stages III and IV PDAC patients.

**Method:**

In this study, weighted gene co-expression network (WGCNA) analysis and a deconvolution algorithm (CIBERSORT) that evaluated the cellular constituent of immune cells were used to evaluate PDAC expression data from the GEO (Gene Expression Omnibus) datasets, and identify modules related to CD4+ T cells. LASSO Cox regression analysis and Kaplan–Meier curve were applied to select and build prognostic multi-gene signature in TCGA Stages III and IV PDAC patients (N = 126). This was followed by independent Stages III and IV validation of the gene signature in the International Cancer Genome Consortium (ICGC, N = 62) and the Fudan University Shanghai Cancer Center (FUSCC, N = 42) cohort. Inherited germline mutations and tumor immunity exploration were applied to elucidate the molecular mechanisms in PDAC. Univariate and Multivariate Cox regression analyses were applied to verify the independent prognostic factors. Finally, a prognostic nomogram was created according to the TCGA-PDAC dataset.

**Results:**

A four-gene signature comprising NAPSB, ZNF831, CXCL9 and PYHIN1 was established to predict overall survival of PDAC. This signature also robustly predicted survival in two independent validation cohorts. The four-gene signature could divide patients into high and low-risk groups with disparity overall survival verified by a Log-rank test. Expression of four genes positively correlated with immunosuppression activity (PD-L1 and PD1). Immune-related genes nomogram and corresponding calibration curves showed significant performance for predicting 3-year survival in TCGA-PDAC dataset.

**Conclusion:**

We constructed a novel four-gene signature to predict the prognosis of Stages III and IV PDAC patients by applying WGCNA and CIBERSORT algorithm scoring to transcriptome data different from traditional methods of filtrating for differential genes in cancer and healthy tissues. The findings may provide reference to predict survival and was beneficial to individualized management for advanced PDAC patients.

## Introduction

Pancreatic ductal adenocarcinoma (PDAC) is one of the most devastating human invasive solid malignancies in the world. Because of its early metastasis and chemotherapy-resistant, the 5-year survival rate of PDAC is less than 5% (1). However, PDAC patients of the same TNM stage may differ in survival, perhaps by reason of the complex immune microenvironment and the of PDAC. Thus, a better understanding of the pathogenesis of cancer and better signatures to help predict prognosis are imperative for improving individualized treatment for PDAC patients.

In recent decades, advances in high-throughput techniques have provided scientists with new insight into PDAC. Yan et al. revealed a four-gene signature (with LYRM1, KNTC1, IGF2BP2, and CDC6) that predicts OS (overall survival) from a PDAC dataset in TCGA using Cox proportional hazards regression analysis (2). Raman et al. developed a five-gene prognostic model that significantly related to the progression of pancreatic cancer through the same method (3). However, on account of the barrier of overfitting in high-dimensional microarray data, this method is not appropriate at some time. Least Absolute Shrinkage and Selection Operator (LASSO) regression could make up for the defects and has been widely used for optimize selection of genes (4). Additionally, PDAC is highly heterogeneous and the pathological characteristics of PDAC with different stages are quite different. Compared to Stage I patients, conventional chemotherapy and cancer immunotherapies have become the standard for first-line treatment of advanced PDAC patients. However, there was no particularity molecular markers for immunotherapy of Stages III and IV PDAC patients. Therefore, a more accurate prognosis immune-related molecular markers for PDAC patients is important to direct better management strategies.

In this study, we explore the effect of the tumor immune microenvironment and performed a systematic and comprehensive gene signature of PDAC. Weighted gene co-expression network analysis (WGCNA) was performed using PDAC Gene Expression Omnibus (GEO) gene expression data (5). The T-cell compositions of samples were estimated by the Cell type Identification by Estimating Relative Subsets ff RNA Transcripts (CIBERSORT) algorithm (6). A four-gene signature was constructed to predict the prognosis of Stages III and IV PDAC patients using the LASSO Cox regression model and was validated in two independent validation cohorts including the Fudan University Shanghai Cancer Center (FUSCC) and the International Cancer Genome Consortium (ICGC). A prognostic nomogram incorporating the gene signature and clinical prognostic factors was established to predict 3-year survival in advanced PDAC patients.

## Material and Methods

### Collection of Genomic Data

We downloaded the PDAC RNA expression data from the Gene Expression Omnibus (GEO, http://www.ncbi.nlm.nih.gov/geo/), which contain data related to 36 tumor samples. The dataset of GSE16515 was obtained applying the platform Affymetrix Human Genome U133 Plus 2.0 Array (HG U133 Plus 2.0). R package “limma” was used to normalize the RNA sequencing data (7). A little variation of sequence data often represents noise, so we employed Coefficient of Variation values to screen the most variant genes, which were then applied to construct the network. 

### Evaluation of Tumor-Infiltrating Immune Cells

In this study, the R package “CIBERSORT” was utilized to estimate the fraction of immune cells of GSE16515 samples. Specifically, the CIBERSORT algorithm was applied to assess the fractions of the 22 types of tumor‐infiltrating immune cells (TIICs). The CIBERSORT is thought to be better than previous deconvolution methods for analysis of unrevealed mixture content and noise. This algorithm could be drawn to estimate the relative composition of cell subpopulations from complex tissue expression profiles, making it a practical tool to estimate the abundances of special cells in intricate tissue.

### Co-Expression Network Construction

Expression values of 2,537 genes were put into construct a weight co-expression network utilizing the R package “WGCNA” (5). First, according to the Pearson’s correlation value among paired genes, the expression levels of microarray data were converted into similarity matrixes. Next, the similarity matrixes were reconstructed to adjacency matrix, on the basis of amn = |cmn| β (cmn = Pearson’s correlation between paired genes; amn = adjacency between paired genes). To classify genes with comparable expression patterns into different modules, we applied a dynamic hybrid cutting method, applying a bottom-up algorithm with a module least value cutoff of 30.

### Construct Module Trait Relationships

Module eigengenes were used to carry out component analysis of each module. We judge the correlation between module eigengenes and the infiltration level of T cells to conclude the significance of modules by Pearson test. We picked the interest T cell subtype and module with the highest correlation coefficient and deemed that as a hub module when *P <*0.05.

### Bioinformatic Analysis

The web tool “Metascape” (http://metascape.org) for enrichment analysis was applied to recognize the function of genes in the hub module (8). The top 20 enriched terms were displayed as a bar graph. Aiming to investigate the relationship between terms, a network graph was presented by similarity greater than 0.3 terms.

### Identification of Hub Genes

Candidate hub genes were selected determined by the modular connectivity and relationship of gene in the hub module. Module connectivity and is clinical trait relationship identified as the absolute value of the Pearson’s correlation (Module Membership) and gene and the trait (Gene Significance). We set the Module Membership >0.6 and the Gene-Significance >0.3 for candidate hub genes. Moreover, we selected genes in the hub module and applied the Search Tool for the Retrieval of Interacting Genes (STRING; https://string-db.org/) online database to construct PPI network and looked for central nodes (9). Genes with a combined score of >0.4 and node connectivity of > 15 were recognized as central nodes. Cytoscape, a free bioinformatics platform, was used to visualize the network (https://cytoscape.org/) (10).

### Establishment of the LASSO Regression Model and Calculation of Risk Score

The candidate genes selected by Venn analysis to compare candidate hub genes and central nodes in the PPI network were selected to construct LASSO Cox regression analysis models with the R package “glmnet”. The “glmnet” package returned a sequence of models (11). For each model, the tuning parameter λ was conversely related with the complexity of the model and the value of deviance. Kaplan–Meier analysis were employed to calculate predictive differences between the high- and low-risk groups based on a cutoff median risk score in the discovery and other two validation datasets. Additionally, a nomogram and corresponding calibration curves were built according to the TCGA cohort for clinical application.

### RNA Extraction, Reverse Transcription, and qRT-PCR Analysis

Total RNA was acquired from 42 patient were diagnosed as Stages III and IV PDAC samples at the FUSCC by using TRIzol Reagent (Invitrogen, USA). TaKaRa PrimeScript RT Reagent Kit (TaKaRa, Japan) was adopted to reverse transcription. ABI 7900HT Real-Time PCR System (Applied Biosystems, USA) was performed to detect the expression of candidate genes. The primers verified in this study are shown in **Supplementary Table S1**.

### Statistical Analysis

Statistical analysis was performed in R (version 3.6.2, www.r-project.org). Spearman correlation analysis was used to determine the association of gene signature expression with PD1 and PDL1 expression. Calibration plots were produced to assess whether actual outcomes matched predicted outcomes for the nomogram.

## Results

### RNA Expression Data

**Figure 1:**
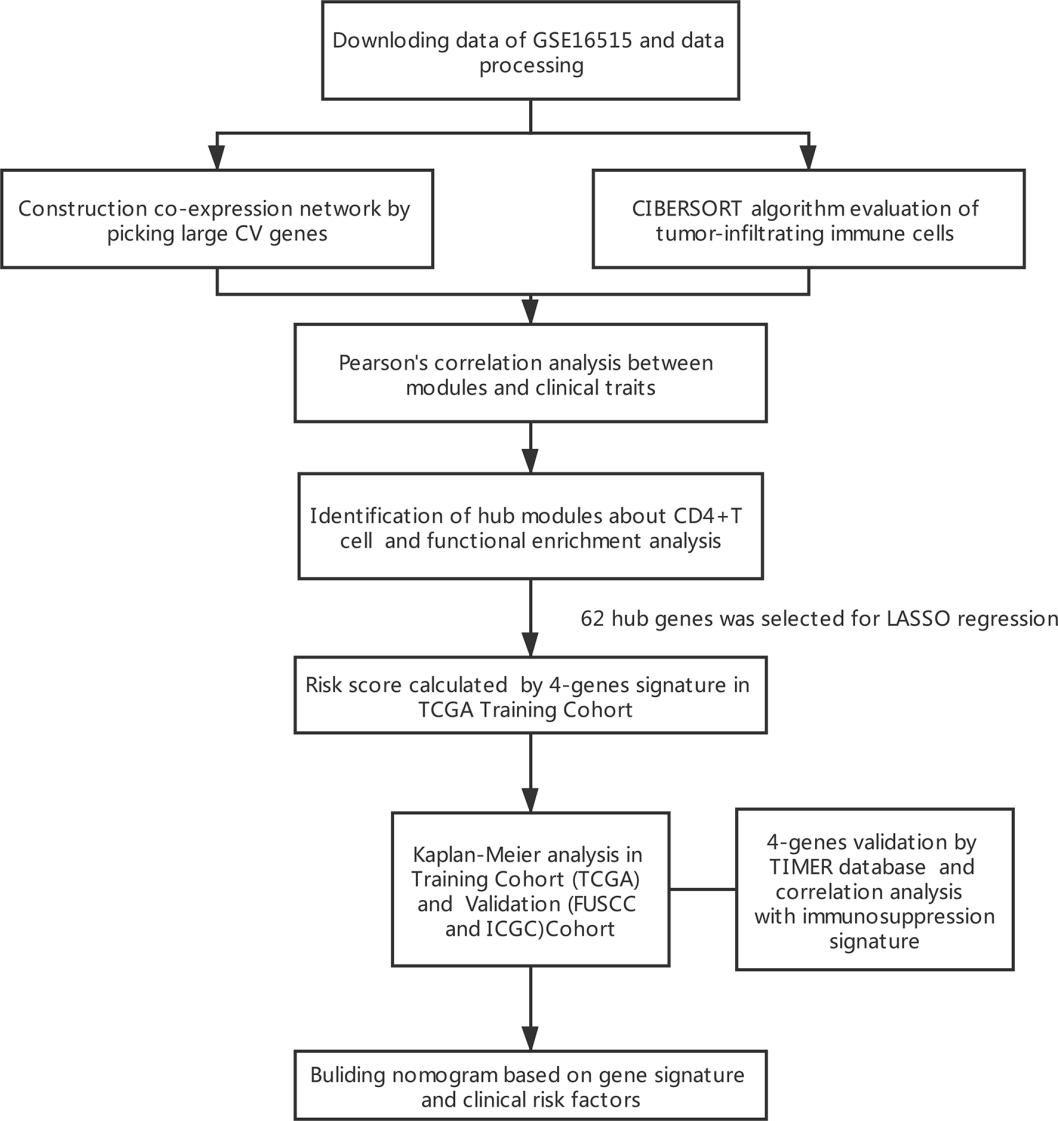
Flowchart presenting the process of establishing the gene signature in this study.

**Figure 2:**
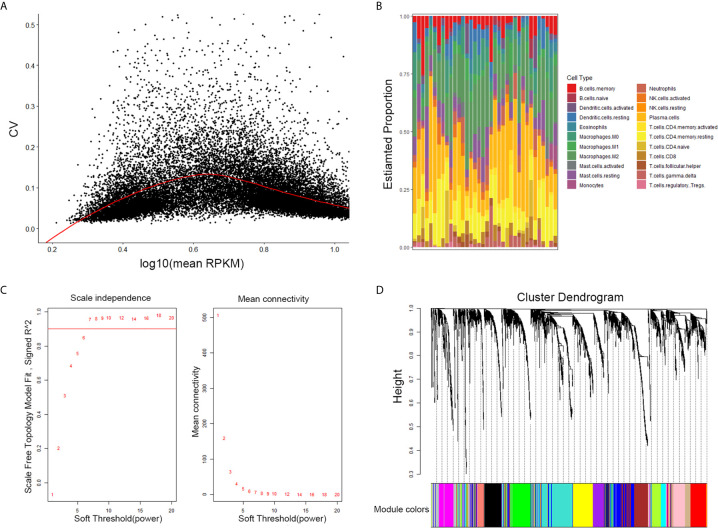
Selection of the appropriate beta value to construct a hierarchical cluster number. **(A)** Selected genes with Coefficient of variation values greater than 0.15. **(B)** Estimate fraction of immune cells by CIBERSORT algorithm in GSE16515. **(C)** Analysis of the scale-free fit index and of the average connectivity of 1–20 soft threshold power. **(D)** Genes are grouped into diverse modules by hierarchical clustering. Different colors represent different modules.

The results of this study are summarized in a flow chart (**Figure 1**). We obtained RNA expression data of 36 PDAC samples in the Gene Expression Omnibus (GEO) database. The median ranking value was used as the expression value if several probes matched a single gene. Approximately 2,568 genes with Coefficient of variation values greater than 0.15 were chosen for the following analysis (**Figure 2A**).

### The CIBERSORT Algorithm Evaluation of Tumor-Infiltrating Immune Cells

CIBERSORT is a systematic algorithm that analyzes RNA expression data to estimate the abundance of various cell subtypes for each sample. The fractions of 22 TIICs were evaluated by the R package “CIBERSORT”. CD8+ T cell, naive CD4+ T, memory resting CD4 + T cells, activated memory CD4+ T cells were accounted for a large proportion of PDAC samples of immune cell infiltration. Then, four subtypes of T cells of 22 TIICs in tumor sample were chosen as trait data of WGCNA (**Figure 2B**).

### Gene Co-Expression Network of PDAC

The expression values of the 2,568 genes were applied to build a co-expression network by the R package “WGCNA” We estimated average linkage and Pearson’s relation values to cluster the samples of GSE16515. Soft threshold power analysis revealed the scale-free fit index of the network topology in the WGCNA pipeline. The optimal soft threshold was 7, where the fitting curve approached 0.9 (**Figure 2C**). Dynamic hybrid cutting was adopted to construct hierarchical clustering tree. Each leaf on the tree shows an independent single gene and genes with similar expression data are close together and form a branch of the tree which represent the gene module (**Figure 2D**).

### Identification of Hub Modules and Functional Enrichment Analysis

**Figure 3:**
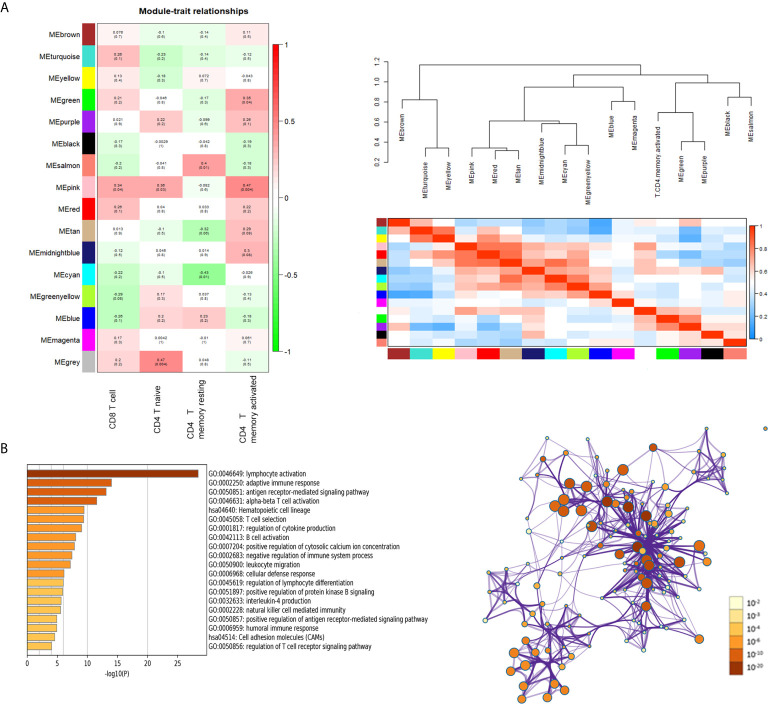
Key modules and feature notes. **(A)** Heatmap and Eigengene-dendrogram show the correlations of module eigengenes with T-cell infiltration. **(B)** The first 20 enriched terms are shown as a bar chart on the left. The protein–protein interaction networks diagram on the right is constructed with each enrichment term as a node and which colored by different cluster ID.

Heatmap of module–trait relationships showing the correlations between module eigengenes and TIICs profile traits. Among the modules, the pink module was highly associated with T cells CD8 (CD8+ T cells) (R^2^ = 0.34, *P* = 0.04), T cells CD4 naïve (R^2^ = 0.36, *P* = 0.03) and T cells CD4 memory activated (R^2^ = 0.47, *P* = 0.004). We were focused specifically on the CD4+ T cells, so the pink module was identified as a hub module due to the high correlation with CD4+ T cells (**Figure 3A**). Genes consisting of pink module were taken into the next analyzation for pathway and process enrichment by the web tool “Matascape”. The 20 highest representative enrichment terms were all immune-related terms, and the four most highly enriched terms were lymphocyte activation, adaptive immune response, antigen receptor-mediated signaling pathway and alpha-beta T cell activation. We then chose a subset of representative terms from this cluster and transformed them into a network layout (**Figure 3B**).

### Identification Hub Genes and Establish Prognostic Signature

**Figure 4:**
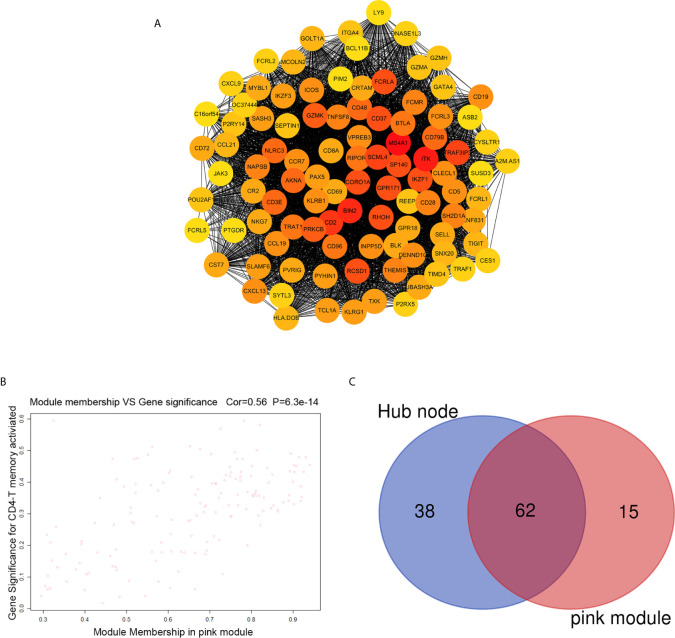
Identification of hub genes. **(A)** PPI network of top 100 genes was selected from the pink module. **(B)** A scatter plot of the genes in the pink module. **(C)** Hub genes were selected according to the overlap between PPI and pink module in co-expression networks.

**Figure 5:**
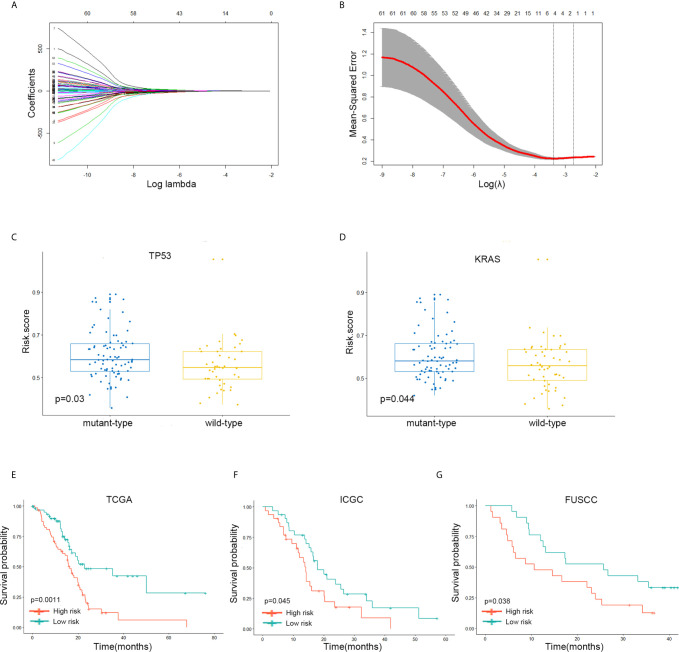
A four-gene signature system was established to predict the overall survival of Stages III and IV PDAC patients in the TCGA discovery and two independent validation cohorts. **(A, B)** LASSO coefficient profiles of the 62 immune-related’ genes. LASSO, least absolute shrinkage and selection operator method. **(C)** The expression level of the risk score in mutation status of TP53 in Stages III and IV PDAC patients of TCGA dataset. **(D)** The expression level of the risk score in mutation status of KRAS in Stages III and IV PDAC patients of TCGA dataset. **(E)** The Kaplan–Meier plot of 5-year overall survival in TCGA Stages III and IV PDAC cohort. **(F)** The Kaplan–Meier plot of 5-year overall survival in ICGC Stages III and IV PDAC cohort. **(G)** The Kaplan–Meier plot of 5-year overall survival in FUSCC Stages III and IV PDAC cohort.

The highly connected genes consist of pink module were investigated as key factors associated with CD4+ T cell infiltration level. From the protein–protein interactions (PPI) network, the top 100 genes in pink module were selected as central nodes according to Degree rich integrals built using the String database and we visualized these results as network layout using Cytoscape (**Figure 4A**). Furthermore, according to the cut-off standard (Module Membership >0.6 and Gene-Significance >0.3), 77 genes in pink module met these criteria and were selected as candidate hub genes (**Figure 4B**). Finally, 62 genes were selected in both analyses by Venn analysis and designated as hub genes (**Figure 4C**). The LASSO coefficient profiles of the 62 genes are presented in **Figure 5**. The LASSO risk score was obtained as follows:

$$\begin{array}{c} \text{Risk score}=(0.32605651\times \text{expression level of CXCL}9)-(0.03660404\times \mathrm{expression level of NAPSB})\\ -(0.07097911\times \mathrm{expression level of PYHIN}1)-(0.24647254\times \mathrm{expression level of ZNF}831) \end{array}$$

The samples with low-risk scores showed better overall survival (OS) times than those with high-risk group patients in the TCGA discovery cohort (*P* = 0.0011; **Figure 5E**). The finding was subsequently validated in ICGC (*P* = 0.045; **Figure 5F**) and FUSCC (*P* = 0.038; **Figure 5G**) validation datasets.

### Validation of the Expression of and Immunosuppression Alterations in the Four Genes

**Figure 6:**
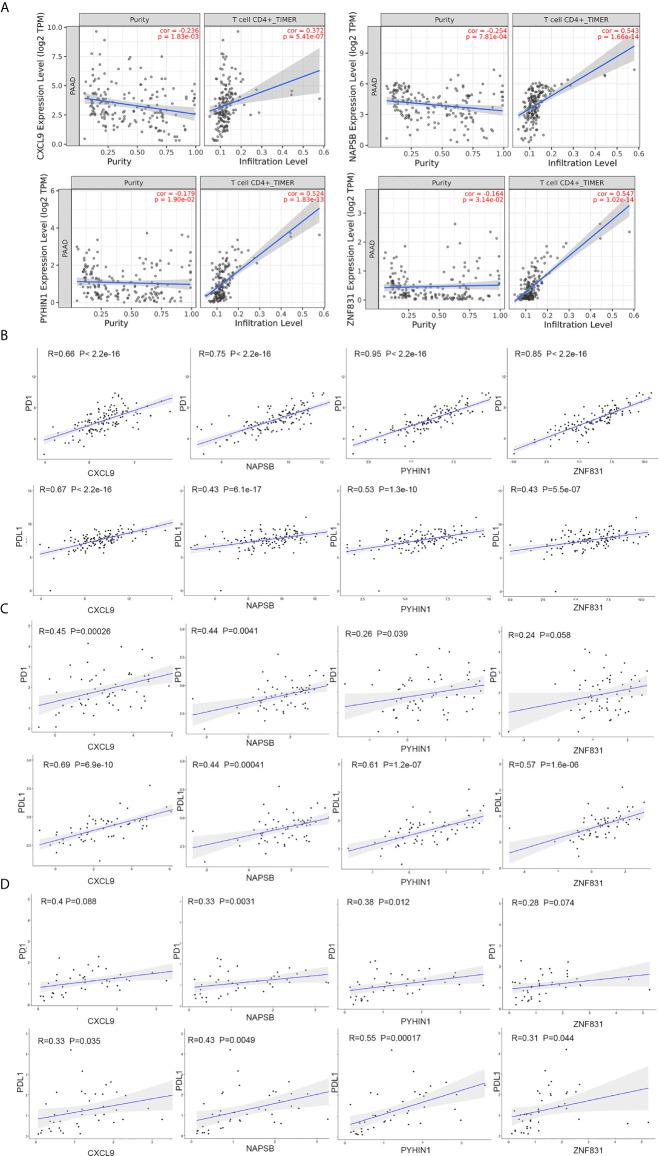
NAPSB, ZNF831, CXCL9 and PYHIN1 associated with CD4+ T lymphocyte infiltration and immunosuppression markers. **(A)** The Timer web tool was performed to estimate the association between the expression levels of four genes with the infiltration level of CD4+ T immune cells in PDAC samples. **(B)** Correlation between four-gene expression and PD1 and PDL1 in PDAC samples of TCGA dataset. Top of Scatter plots depicts R2 and p-values. **(C)** Correlation between four-gene expression and PD1 and PDL1 in PDAC samples of ICGC dataset. **(D)** Correlation between four-gene expression and PD1 and PDL1in PDAC samples of FUSCC dataset.

To explore the association between these hub genes and CD4+ T cells, we investigated the expression data for four genes in the TIMER database. The results revealed positive correlation of the expression values of the four genes with the infiltration levels of CD4+ T cells (**Figure 6A**). To investigate the relation between the expression of four genes and immunosuppression markers (PD1 and PDL1), we use the expression data obtained from the TCGA and ICGC database to validate the correlation test. Furthermore, we collected 42 patients’ samples from our center (FUSCC) to test if these expression of four genes was associated with PD1 and PDL1 expression. Taking an unbiased approach, we found that four genes showed a positive and significant correlation with PD1 and PDL1counts in three databases (**Figures 6B–D**).

### Correlations Between the Three-Gene Signature and Clinical Characteristics

**Figure 7:**
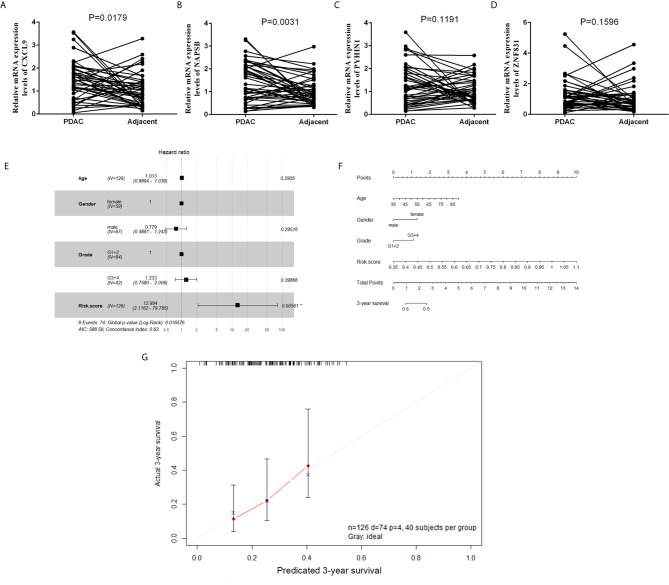
The expression and clinical significance of gene signature in PDAC. **(A–D)** Expression of four genes in tumor and adjacent normal tissues from a cohort of 42 PDAC patients was determined by qPCR. **(E)** Forest plot summary of multivariable Cox regression analyses of the risk score, age, gender and grade on TCGA cohort. The squares represent the hazard ratio (HR), and the transverse lines represent 95% CI. CI, confidence interval. **(F)** A nomogram to predict survival probability at 3-year for PDAC patients based on the results deriving from the TCGA cohort. **(G)** Calibration curve for the nomogram when predicting 3-year overall survival.

To validate the reliability of the results of the risk‐score of four genes (NAPSB, ZNF831, CXCL9, PYHIN1), their actual expression of 42 PDAC samples were examined with quantitative reverse‐transcription polymerase chain reaction (qRT‐PCR). The results showed that NAPSB and CXCL9 were upregulated in PDAC tumor tissues with statistical significance (**Figures 7A–D**). Univariate and multivariate Cox proportional hazards regression showed risk score could predict poor survival of PDAC patients, as shown in **Supplementary Table S2** and **Figure 7E** (*P* = 0.0013; 0.00561). The patients from the TCGA dataset were used to establish a prognostic nomogram predicting a 3-year overall survival probability in PDAC patients according to the stepwise Cox regression model (**Figure 7F**). Calibration plots showed that the nomogram presented good agreement at predicting overall survival and the actual proportion in pancreatic cancer patients (**Figure 7G**).

## Discussion

PDAC remains a major cause of cancer-related death worldwide, and the incidence and mortality are estimated to increase substantially by 2030 (12). Several studies have reported signatures that could effectively predict overall patient survival, including a five-miRNA signature (13), and a 3-lncRNA signature (14). There are less models that focus on Stages III and IV patients, and the prognosis is rather heterogeneous for these group. In addition, these commonly-used models only incorporate clinical and pathological factors, without considering the role of immune cells from the tumor microenvironment play in tumor progression and prognosis of PDAC patients. Thus, a more precise prognosis model for advanced patients is necessary.

With the rapid development of bioinformatics technology, many tools have been developed to find biomarkers (15). The CIBERSORT is a deconvolution algorithm to quantify the cellular composition of immune cells, such as prostate cancer and kidney cancer (16, 17). The WGCNA is another bioinformatics tool that can be used to recognize correlation modules and hub genes for cancer. Immune checkpoint inhibitors have shown promising initial efficacy in advanced PDAC. This has increased focus on exploring the potential immune-related factors for immunotherapy. CD4+ T cells play a key role in immunotherapy. In the current study, a four-gene signature correlated to CD4+ T cell infiltration level was identified by utilizing the LASSO Cox regression model. In this study, we utilized WGCNA and CIBERSORT algorithms to perform gene expression matrix to establish the co-expression network and estimated the infiltration level of T cells by, and interactions were adopted to identify the genes most related to CD4+ T cells. The gene enrichment analysis of the selected hub module also proves that it is a highly immune-related module. Based on this information, a four-gene signature correlated to CD4+ T cell infiltration level was identified by utilizing the LASSO Cox regression model. Querying the relationship between these four-genes (NAPSB, ZNF831, CXCL9 and PYHIN1) and immune cells in the TIMER database revealed positively correlated expression of these genes with CD4+ T cells. Positive correlation between four-gene expression and immunosuppression markers (PD1 and PDL1) was proved in the TCGA, ICGC and FUSCC datasets.

Chemokines are a group of small cytokines that can cause directed chemotaxis. Secreted by various types of cells including inflammatory macrophages dendritic cells (particularly the cDC1 subtype), endothelial cells, fibroblasts, and tumor cells themselves, chemokines could be categorized based on their behaviors and structure characteristics (18). The CXC chemokines are a family of 17 α-chemokines that carried out multiple pathological or physiological functions. The role of CXCL9 play in human cancers remains ambiguous and contradictory. It had been found that CXCL9 is expressed in most types of human cancers, such as hepatocellular carcinoma, melanoma, gastric carcinoma, cervical cancer, as well as PDAC (18). Some previous studies have delineated that CXCL9 could be presented as both having promoting and suppressing effects on CD8+ cytotoxic T cells. In prostate tumors it was found out that there is strong positive correlation between CXCL9 and CD8 expression (19). Chow et al. also proved that CXCL9 facilitated the dendritic cell-CTLs interaction and activated the CD8+ T cells response in the tumor microenvironment (20). Additionally, a recent report by Gao et al. shows that CXCL9 activated STAT3 signaling in CD8+ T cells of PDAC cell, and suppression of STAT3 could recover the proliferation and secretion of anti-tumor cytokines of CD8+ T cells (21). The PYHIN1 (Pyrin and HIN Domain Family, Member 1) gene is predicted to encode six different protein isoforms as a result of alternative mRNA splicing (a1, a2, b1, b2, g1, and g2). Each of the isoforms has a common N-terminal region, which contains a PYD and an NLS. Functionally, roles for PYHIN1 in controlling cell cycle, differentiation, and apoptosis growth have been reported (22). PYHIN1 expression has been presented to be reduced in breast tumors. Recent studies have delineated that PYHIN1a1 controls tumor suppressive activity mediated by the destabilization of the oncoprotein HDM2 (23). Information about ZNF831 and NAPSB is scarce in the literature.

## Conclusion

In conclusion, our study successfully identified potential CD4+ T cell related biomarkers and a prognostic nomogram incorporating the gene signature and clinical prognostic factors for prediction of OS in Stages III and IV PDAC patients. Novel insights into the relationship between immune and PDAC were shown in this study. Future investigations on the molecular mechanisms and prospective randomized clinical trials will bring a roadmap for precision medicine.

## Data Availability Statement

The original contributions presented in the study are included in the article/**Supplementary Material**. Further inquiries can be directed to the corresponding authors.

## Ethics Statement

The studies involving human participants were reviewed and approved by ethical code: 050432-4-1212B. The patients/participants provided their written informed consent to participate in this study.

## Author Contributions

CL and JX designed this study; ZT performed the bioinformatics analyses and wrote the manuscript; YL and BZ analysis and interpretation of data; SS, JL and XY statistical analysis; CL and JX supervised this study. All authors contributed to the article and approved the submitted version.

## Funding

This study was jointly funded by the national natural Science Foundation of china (No. 81902428, 81802352 and 81772555), the Shanghai Sailing Program (No. 19YF1409400), the national Science Foundation for Distinguished Young Scholars of china (No. 81625016), Clinical and Scientific Innovation Project of Shanghai Hospital Development Center (SHDC12018109), and Scientific Innovation Project of Shanghai Education Committee (2019-01-07-00-07-E00057).

## Conflict of Interest

The authors declare that the research was conducted in the absence of any commercial or financial relationships that could be construed as a potential conflict of interest.

The reviewer CJ declared a shared affiliation, with no collaboration, with the authors, to the handling editor at the time of the review.

## Publisher’s Note

All claims expressed in this article are solely those of the authors and do not necessarily represent those of their affiliated organizations, or those of the publisher, the editors and the reviewers. Any product that may be evaluated in this article, or claim that may be made by its manufacturer, is not guaranteed or endorsed by the publisher.
