# Supplementary material for: Analysis of Immune-Related Signatures Related to CD4+ T Cell Infiltration With Gene Co-Expression Network in Pancreatic Adenocarcinoma
Source: Front Oncol. 2021 Jul 23;11:674897. doi: 10.3389/fonc.2021.674897 (PMC8343184; doi:10.3389/fonc.2021.674897)
Supplement: Supplementary file 1 [file Table_1.docx]

The sequences of the primers for the three genes signatures.

| Primer | Sequence (5'to 3') | Base number |
| --- | --- | --- |
| m-CXCL9-F | CCAGTAGTGAGAAAGGGTCGC | 21 |
| m-CXCL9-R | AGGGCTTGGGGCAAATTGTT | 20 |
| m-NAPSB-F | CCACCGCTTCAATCCCAATG | 20 |
| m-NAPSB-R | AGCTTCCCCGAAAATCACGG | 20 |
| m-PYHIN1-F | CCAAGCAACCGTCTCACAG | 19 |
| m-PYHIN1-R | GCCGAGTCTGCTCTTTGGA | 19 |
| m-ZNF831-F | CTAGTGACGGGCAGCCTAGAT | 21 |
| m-ZNF831-R | TGCGGACACAGGTACTTGC | 19 |
| m-CD274 -F | TGGCATTTGCTGAACGCATTT | 21 |
| m-CD274 -R | TGCAGCCAGGTCTAATTGTTTT | 22 |
| m-PDCD1-F | CCAGGATGGTTCTTAGACTCCC | 22 |
| m-PDCD1-R | TTTAGCACGAAGCTCTCCGAT | 21 |
