# Supplementary material for: Analysis of Immune-Related Signatures Related to CD4+ T Cell Infiltration With Gene Co-Expression Network in Pancreatic Adenocarcinoma
Source: Front Oncol. 2021 Jul 23;11:674897. doi: 10.3389/fonc.2021.674897 (PMC8343184; doi:10.3389/fonc.2021.674897)
Supplement: Supplementary file 2 [file Table_2.docx]

Univariable Cox regression analyses of the risk score, age, gender and grade in TCGA.

|  | HR (95% CI for HR) | Wald.test | P.value |
| --- | --- | --- | --- |
| Age | "1 (1-1)" | "2.9" | "0.09" |
| Gender | "0.75 (0.47-1.2)" | "1.5" | "0.22" |
| Risk. score | "17 (3-100)" | "10" | "0.0013" |
| Grade | "1.3 (0.8-2.1)" | "1.1" | "0.29" |
